# How to Feed the Sick

**Published:** 1880-09

**Authors:** E. Ingals

**Affiliations:** Chicago


### ARTICLE VI.

### HOW TO FEED THE SICK. By E. ENGALS, M. D., Chicago.

Dr. Tanner's experiment of long abstinence from food has attracted much attention, and, judging from the tone of the daily press, I think the public deem it an idle and foolish exhibition, but in its scientific aspect it has great interest, and will doubtless be repeated by others, and we shall have champion fasters as we now have champion rowers and athletes of the diamond field and prize ring. From this we may come to give due weight to the capacity which the body has to feed upon itself, and this will lessen the fear that patients will starve, and will help us to wait patiently for physiological repair of pathological changes. It is often remarked that truth is apt to be midway between extremes; yet public opinion goes from one extreme to another, and though we are conscious of our liability to this fault, we are generally ready to follow the pendulum through the entire arc of its motion. Within my memory the common practice was to interdict even a cup of cold water from the parched lips of a fever patient famishing with thirst. After the favorable termination of a protracted disease, when the attenuated body was clamoring for nutrition to restore what had been lost, food would be withheld to a degree that much prolonged the period of convalescence. Then came a change, and the direction of all in authority was that the sick should be "fed well." This was called "supporting treatment,"

and as all knew that the body was sustained by nutrition, the practice strongly addressed itself to both public and professional favor. As a consequence patients who loathed even the thought of food and abhorred its sight, were compelled to take it in considerable quantities at frequent and stated intervals, as if it were a medicinal dose. Under such circumstances food is not well digested, but is partially decomposed in the alimentary canal, and some products of such decomposition may be taken up by the absorbents to poison the blood, and the debris while retained disturbs the whole system and is thrown off by the stomach and bowels with difficulty. In ordinary sickness it is best that the patient should order his own diet list, and for the physician to expunge from this what he judges may prove harmful. If the patient does not desire food it is generally better to leave him without it, and I can attest, from frequent observation, that this may be done with entire safety until a condition of extreme exhaustion has been reached, and even then, if the patient's desire for food does not return, stimulants are oftener indicated medicinally than nutrients. We should not forget that in sickness the digestive organs are enfeebled as well as the nervous and muscular, and their labors should be proportionately lightened. It is many years since I have seen a sick person injured from having taken too little food, while I have seen many who have suffered from taking too much.

---

WE hasten to correct an oversight that occurred in the last issue of the JOURNAL, in that we failed to give the credit of the admirable selection, "On the Summer Complaint of Infants," by J. Lewis Smith, M.D., to our valuable exchange, the *Medical News and Abstract.*

DR. J. N. HYDE of the editorial corps is absent, attending the meeting of the Dermatological society, and when he returns he will give our readers a full report of the meeting of that society.

DR. F. C. HOTZ, our old associate, has rendered us much assistance in making up this number of the JOURNAL.

